# 体积排阻色谱法定量检测9种型别人乳头瘤病毒原液中病毒样颗粒

**DOI:** 10.3724/SP.J.1123.2020.06032

**Published:** 2021-04-08

**Authors:** Zhen LONG, Xiaoyu LI, Xiuling LI, Junkai LIU, Jianhui NIE, Changkun LI, Yueqi LI, Taohong HUANG, Weijin HUANG

**Affiliations:** 1.岛津企业管理(中国)有限公司, 北京 100020; 1. Shimadzu (China) Co., LTD., Beijing 100020, China; 2.中国食品药品检定研究院, 卫生健康委员会生物技术产品检定方法及其标准化重点实验室, 国家药品监督管理局生物制品质量研究与评价重点实验室, 北京 102629; 2. National Institutes for Food and Drug Control, Key Laboratory of National Health Commission for Research on Quality and Standardization of Biotech Products, Key Laboratory of Biological Product Quality Research and Evaluation of National Medical Products Administration, Beijing 102629, China; 3.中国科学院分离分析化学重点实验室, 中国科学院大连化学物理研究所, 辽宁 大连 116023; 3. Key Laboratory of Separation Science for Analytical Chemistry, Dalian Institute of Chemical Physics, Chinese Academy of Sciences, Dalian 116023, China

**Keywords:** 体积排阻色谱, 定量, 人乳头瘤病毒, 病毒样颗粒, 疫苗, size exclusion chromatography (SEC), quantitative analysis, human papilloma virus (HPV), virus-like particle (VLP), vaccine

## Abstract

据统计,5%以上的人类癌症由人乳头瘤病毒(HPV)导致。HPV疫苗的使用,尤其是多价HPV疫苗的使用,可有效预防HPV感染和肿瘤的发生。例如,9价HPV疫苗可有效预防90%以上HPV相关癌前病变。人乳头瘤病毒样颗粒(VLP)是HPV疫苗的唯一抗原。VLP由360份衣壳蛋白L1组成。VLP的含量测定对HPV原液和HPV疫苗的质量评价至关重要。该文发展了一种以体积排阻色谱(SEC)为基础的9种型别人乳头病毒样颗粒的定量方法。实验优化了包括色谱柱类型、色谱柱孔径、流动相离子强度和流动相pH值在内的色谱条件。经过考察,以SHIMSEN Ankylo SEC-300色谱柱(300 mm×7.8 mm, 3 μm)为固定相,以含有300 mmol/L NaCl和50 mmol/L磷酸盐(pH 7.0)的缓冲溶液为流动相时,VLP的色谱峰更窄,从而可获得更高的响应和更好的灵敏度,因此选择该色谱条件用于VLP与基质的分离。优化所得的方法具有较宽的线性范围,良好的重复性(峰面积的相对标准偏差不大于5.0%)和灵敏度(定量限为4.58~15.24 μg/mL)。将方法用于HPV原液中VLP的含量测定,监测VLP的稳定性。结果显示,HPV原液中VLP颗粒不稳定,于4 ℃放置一周后,VLP含量与生产后立即测得的含量相比存在一定程度的降解。此外,方法还可用于疫苗上清液中游离蛋白质的分析,监测铝佐剂对VLP的吸附情况。被测厂家的铝佐剂可较好的吸附VLP,无明显残余蛋白质检出。与传统的蛋白质定量方法相比,如Folin-酚法(Lowry法),该法具有操作简单、自动化程度高、分析通量高等优点,可实现VLP含量的批量化分析。

## Abstract (en)

Cervical cancer is the fourth most common cancer among women. Human papilloma virus (HPV) is the most common cause of cervical cancer which accounts for 5% of all human cancers and results in about 528000 cases and 266000 deaths every year. HPV vaccines are considered the most effective strategy for the prevention of HPV infection and cervical carcinoma. Since 2006, three prophylactic vaccines against HPV have been available on the market, including bivalent vaccines, quadrivalent vaccines, and nine-valent vaccines. Among them, nine-valent vaccines have been reported to be the most effective. They can prevent 97% of the high-grade pre-cancer lesions. Virus-like particles (VLPs), which are arranged as 360 copies of capsid proteins L1, are the only antigens of the HPV vaccine. Nine-valent HPV vaccines are prepared by mixing nine types of VLPs with adjuvants. Thus, the quality of the VLPs, including their stability and content in the HPV bulk, is very important for developing HPV vaccines. In this study, a method was developed for the determination of the nine types of VLPs (HPV6/11/16/18/31/33/45/52/58) in HPV bulk by size exclusion chromatography (SEC). The parameters of this method were optimized in terms of column brand, pore size of stationary phase particles, buffer concentration, and pH value. SHIMSEN Ankylo SEC-300 column (300 mm×7.8 mm, 3 μm) combined with a buffer aqueous solution containing 300 mmol/L NaCl and 50 mmol/L phosphate (pH 7.0) was utilized to separate the VLPs from the matrix since a narrow peak shape and good repeatability for VLPs could be obtained with this column and mobile phase. The optimized method had a wide linear range, good repeatability (RSDs of peak area were not more than 5.0%), and a satisfactory sensitivity (LOQs in the range of 4.58-15.24 μg/mL). The optimized method was used to determine the VLPs in the HPV bulk. The LOQs of the current method were much lower than the content of the nine types of VLPs in the HPV bulk, indicating that this method was sensitive enough for the determination of the nine types of VLPs in the HPV bulk. The method was also used to determine the VLPs in an HPV bulk that had been stored at 4 ℃ for one week. A decrease in the nine types of VLPs in the range of 10.0%-62.6% was observed after they were stored at 4 ℃ for one week. An HPV vaccine was prepared by mixing the VLPs with an adjuvant. Thereafter, the VLPs were adsorbed on the surface of the adjuvant. The developed method was applied to determine the free VLPs in twelve batches of HPV vaccines from three different manufacturers. No obvious free protein was detected in the twelve batches of the HPV vaccines from the three manufacturers, indicating that VLPs from these manufactures react well with their aluminum adjuvant. Folin-phenol (Lowry assay) is commonly used for the determination of proteins in vaccines. It is based on the reduction of phosphomolybdotungstic mixed acid chromogen in the phosphomolybdotungstic reagent, which results in an absorbance maximum at 650 nm. The Lowry method was sensitive to interfering substances. Most interfering substances caused a lower color yield, while some detergents caused a slight increase in color. To reduce the effect of the interfering substances, a procedure for precipitating the proteins was usually required before the sample was tested. Thus, the Lowry assay is complex, time-consuming, and of low selectivity. Compared to the Lowry method, the method we developed is simpler and more automatic. It is a high-throughput method of determining VLPs. It can be used to determine VLPs in HPV bulk and free VLPs in HPV vaccines.

宫颈癌是威胁女性健康的第四大常见恶性肿瘤类疾病,同时也是女性因癌症而死亡的主要原因之一^[1]^。据统计,每年有45.3万例宫颈癌案例报道^[2]^,该疾病造成每年25万例死亡^[3]^。感染人乳头瘤病毒(HPV)是导致宫颈癌的主要原因,在大约95%的宫颈恶性病变样本中检出人乳头瘤病毒DNA^[4]^。HPV疫苗是预防宫颈癌最有效的手段之一。研究显示,9价HPV疫苗(如Gardasil^®^ 9)可有效预防97%的癌前病变损伤^[5]^。

HPV疫苗由HPV病毒样颗粒(VLP)的原液与适当比例铝佐剂结合制成。HPV成熟的VLP是直径为50~55 nm并具有三角形剖分数(*T*)等于7的20面体对称结构^[6,7]^。HPV疫苗中的VLP由重组技术表达。重组技术表达的VLP是由360份主要衣壳蛋白(L1)单体聚合而成的星状五聚体(即壳粒,capsomere)。HPV病毒衣壳由72个壳粒通过相互作用组装而成,其超微结构和免疫原性与天然HPV病毒类似^[8,9]^。目前上市的HPV疫苗由不同型别的VLP纯化制成。例如,英国葛兰素史克公司生产的HPV疫苗Cerarix^®^由HPV16/18 2种型别的VLP制成;美国默沙东公司HPV疫苗Gardasil^®^ 9由HPV6/11/16/18/31/33/45/52/58 9种型别的VLP制成。在中国,除万泰沧海外,还有9家企业15个品种的HPV疫苗处在研发阶段。

VLP原液的质量(纯度、颗粒度分布、VLP含量等)直接影响HPV疫苗的安全性和有效性。VLP的纯度指VLP原液含有的总蛋白质中VLP所占的质量分数。VLP颗粒大小通常不是一个固定的数值,而是在一定数值范围内。VLP含量是VLP原液中VLP的浓度。Folin-酚法(Lowry法)是疫苗领域常用的蛋白质含量测定方法,可有效实现疫苗原液中总蛋白质含量的测定。但该方法需要复杂的衍生步骤,耗时较长且自动化程度低,无法实现样品的高通量检测^[10,11]^。此外,该方法不仅可以衍生VLP还可以衍生其他具有衍生官能团的小分子物质。因此,检测结果可能会受基质干扰以及衍生效率的影响,从而导致结果偏差。Long等^[12]^发展了基于液相色谱-串联质谱的蛋白质含量测定方法,用于百日咳疫苗中抗原蛋白的含量测定。本实验室^[13]^也发展了基于液相色谱-串联质谱的2价HPV疫苗中HPV16/18抗原蛋白的含量测定。液相色谱-串联质谱法灵敏度高,选择性好,可用于复杂基质中抗原蛋白的含量测定,但前处理时间较长。体积排阻色谱(SEC)具有分离小分子基质和大分子目标化合物的作用,可用于排除小分子基质对大分子目标化合物检测的干扰。在过去几十年间,SEC被用作一种高分子表征技术,如高分子聚合物和蛋白质的分子量分布检测^[14,15]^。该技术也用作HPV原液中VLP颗粒度分布的检测,如Mach等^[16]^采用体积排阻色谱表征VLP颗粒度分布检测以及加热前后VLP的变化情况。现有SEC能有效监测VLP大小和分布是否符合预期,但无法用于VLP含量的测定。本文发展了一种基于SEC的方法,用于HPV疫苗原液中VLP的含量测定以及HPV疫苗中游离VLP的检测。

## 1 实验部分

### 1.1 仪器、试剂与材料

生物惰性液相色谱系统,包括LC-20Ai高压二元泵、脱气机、SIL-20AC自动进样器、CTO-20AC柱温箱、SPD-M20A紫外检测器,所有流路均为聚醚醚酮(PEEK)材质,LabSolutions工作站用于数据处理。以上仪器和软件均购自岛津公司(日本)。Allegra^®^ 64R离心机购自贝克曼公司(美国)。

磷酸氢二钠(98%)、磷酸二氢钠(98%)、磷酸(85%)、氯化钠(99.99%)购自Sigma公司(美国)。低吸附离心管购自Merck公司(美国)。超纯水由Milli-Q系统制备(Millipore公司,美国)。SHIMSEN Ankylo SEC-300色谱柱(300 mm×7.8 mm, 3 μm)购自岛津技迩公司。9个型别的HPV原液(包括HPV6/11/16/18/31/33/45/52/58)、铝佐剂和HPV疫苗由中国食品药品检定研究院提供。HPV疫苗样品由厂家1(4个批次)、厂家2(4个批次)和厂家3(4个批次)提供。HPV原液和HPV疫苗均存储于4 ℃冰箱。

### 1.2 样品前处理

取疫苗样品于低吸附离心管中,以14000 r/min转速离心10 min,取上清液进样分析。

### 1.3 HPLC条件

色谱柱为SHIMSEN Ankylo SEC-300柱(300 mm×7.8 mm, 3 μm);柱温为30 ℃;流动相为含300 mmol/L氯化钠和50 mmol/L磷酸盐(pH 7.0)的水溶液;流速为1 mL/min;检测波长为280 nm;进样体积为10 μL;自动进样器温度为4 ℃。

## 2 结果与讨论

### 2.1 实验条件考察

2.1.1 色谱柱考察

与小分子分析相比,蛋白质等大分子分析对色谱柱的要求更高。除内径、长度、粒径、比表面积、键合相和键合密度等参数以外,键合相和基质之间的亲水层键合工艺以及固定相孔径也对蛋白质的色谱行为有较大影响。由于蛋白质的特殊性质(强疏水性和强电荷性),用于蛋白质分析的体积排阻色谱往往需要在固定相基质和键合相之间涂覆/键合亲水层,用于屏蔽蛋白质与基质之间的非特异性吸附,从而减少蛋白质在色谱柱上的死吸附,提高方法的灵敏度和稳定性。由于亲水层的工艺不同,不同厂家的色谱柱对蛋白质的吸附程度不同。本文考察了两种不同亲水层键合工艺的蛋白质分析SEC柱。最终选择能为考察样品提供较好峰面积重复性的SHIMSEN Ankylo SEC色谱柱进行后续的方法优化。以含量为1276、319、159 μg/mLHPV6的VLP样品为例,采用SHIMSEN Ankylo SEC色谱柱分离分析,每种含量分别进样5次,获得的峰面积RSD均不超过5%。采用相同规格的另一品牌的SEC色谱柱时,峰面积RSD超过20%。

除亲水层的键合工艺,SEC柱的孔径也是影响蛋白质色谱行为的重要因素。本文分别考察了孔径为30、50和100 nm的色谱柱(SHIMSEN Ankylo SEC-300、SEC-500、SEC-1000(300 mm×7.8 mm, 3 μm))。VLP颗粒分布的表征通常使用大孔径SEC柱,如孔径为100 nm的SEC柱^[16]^,如图1所示,HPV6的VLP颗粒在SHIMSEN Ankylo SEC-1000柱上具有较好的分布,在6~11 min期间均有VLP的信号,但色谱峰较宽,导致灵敏度较低,不适合VLP的定量分析。色谱柱SEC-300和SEC-500均可实现VLP与小分子干扰物质的分离,以及VLP的快速洗脱,但SEC-300色谱柱可提供更窄的色谱峰,有利于提高检测灵敏度。因此,以SHIMSEN Ankylo SEC-300柱为固定相,用于HPV的VLP分析。VLP并非单一尺寸的颗粒,在SEC色谱柱上存在一定的颗粒度分布,因此会导致色谱峰并非对称的高斯峰。

**图 1:**
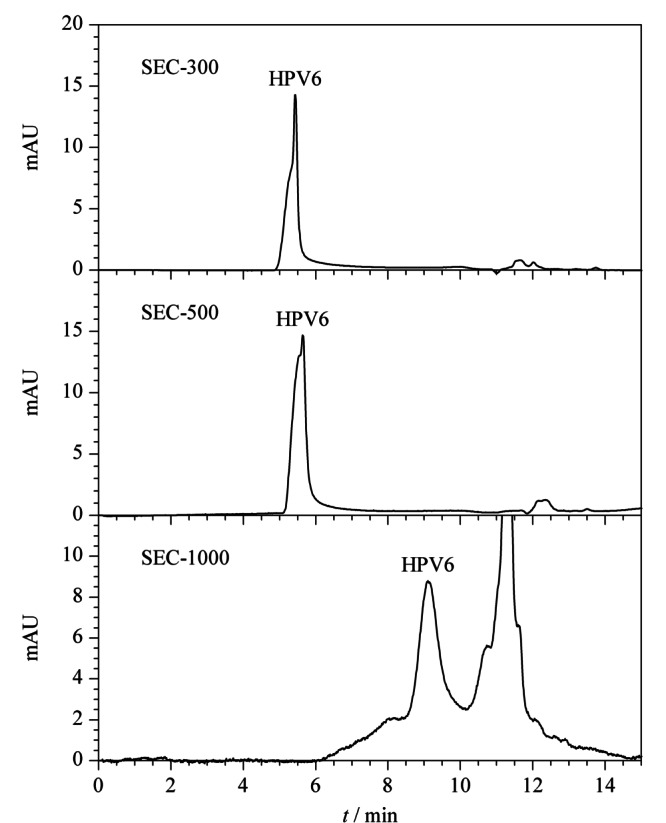
采用SEC-300、SEC-500和SEC-1000柱时HPV6的VLP色谱图

2.1.2 流动相组成考察

离子强度的增加有利于屏蔽蛋白质与色谱柱之间的静电作用,从而改善峰形,减少死吸附和提高响应。本文考察了不同浓度(150、300和500 mmol/L)氯化钠溶液对VLP峰形的影响。固定缓冲盐磷酸盐的浓度(50 mmol/L)和pH值(7.0),随着氯化钠浓度从150 mmol/L增加至300 mmol/L, HPV6峰高随之增加。再增加氯化钠浓度,峰高无显著变化。因此,以300 mmol/L氯化钠(含50 mmol/L pH 7.0的磷酸盐)为流动相。

2.1.3 流动相pH值考察

流动相的pH值会影响固定相和蛋白质的带电情况。本文考察了在流动相pH 3.0和pH 7.0的条件下,VLP的出峰情况。相对于采用pH 3.0的流动相,采用pH 7.0的流动相,VLP响应更强。可能的原因是组成HPV的VLP的L1蛋白等电点(PI)大多在8.0左右。蛋白质PI由在线蛋白质等电点查询软件ProtParam计算(https://web.expasy.org/protparam/)。中性条件下,固定相与蛋白质之间的静电吸引作用减弱,有利于蛋白质的洗脱。

### 2.2 方法学考察

本文从线性范围、检出限(LOD)、定量限(LOQ)和重复性对发展的方法进行了考察。

2.2.1 线性范围

取9种型别HPV的VLP原液,用流动相稀释,在表1浓度范围内配制工作曲线溶液。每种型别的VLP工作曲线不少于5个浓度点,每个浓度样品进样3次。为了更为准确的测得待测样品浓度,本研究线性曲线的最高浓度点和最低浓度点以待测样品浓度为参考进行制定。以每个浓度所得VLP的峰面积与对应浓度进行线性回归,得线性方程见表1。该方法可为9种型别HPV的VLP提供较好的线性,可用于该浓度范围内样品中VLP的含量测定。

**表 1:** 9种VLP颗粒的线性范围、线性方程、相关系数(*R*^2^)、检出限和定量限

VLP	Linear range/(μg/mL)	Linear equation	*R* ^2^	LOD/(μg/mL)	LOQ/(μg/mL)	
HPV6	39.9-1276.1	*A*=203.3*C*-315.7	0.998	2.11	6.38	
HPV11	31.7-629.3	*A*=335.5*C*-290.8	0.998	1.83	5.53	
HPV16	82.3-2632.5	*A*=784.3*C*-168.4	0.997	1.51	4.58	
HPV18	51.2-1639.2	*A*=2173.8*C*-134.0	0.998	3.70	11.20	
HPV31	25.7-822.1	*A*=1266.5*C*-2880.5	0.999	1.75	5.30	
HPV33	23.1-738.1	*A*=1256.8*C*-2996.7	1.000	2.43	7.38	
HPV45	24.0-768.2	*A*=1491.5*C*-439.6	0.999	5.03	15.24	
HPV52	25.5-816.3	*A*=114.3*C*-303.5	0.998	3.83	11.62	
HPV58	24.2-773.2	*A*=929.1*C*-331.8	0.998	3.25	9.84	

*A*: peak area; *C*: mass concentration, μg/mL.

2.2.2 检出限和定量限

以工作曲线的最低浓度点对应的样品为母液,用流动相稀释。当样品所得信噪比接近但不低于3和10时,该浓度作为检出限(LOD)和定量限(LOQ)。9种型别VLP的LOD和LOQ见表1。2.2.3 重复性蛋白质容易吸附在液相色谱的不锈钢管路和色谱柱中,导致峰面积重复性较差。为了避免不锈钢系统对蛋白质的死吸附,本方法所有流路均采用PEEK材质。PEEK材质流路具有蛋白质吸附低、耐受高浓度氯化钠和pH耐受范围宽等优点。本文考察了高、中、低3个水平VLP峰面积的重复性,每种浓度进样5针,以峰面积RSD考察重复性。如表2所示,本方法可为9种型别不同水平HPV的VLP颗粒分析提供较好的重复性,高、中、低3种水平下,峰面积的RSD均不超过5%。表明该方法具有较好的重复性。

**表 2:** 高、中、低水平下VLP峰面积的相对标准偏差

VLP	High level			Medium level			Low level		
	Content/(μg/mL)	RSD/%		Content/(μg/mL)	RSD/%		Content/(μg/mL)	RSD/%	
HPV6	1276.1	1.55		319.0	1.88		159.5	4.45	
HPV11	629.3	2.68		157.3	4.13		78.6	2.81	
HPV16	2632.5	2.37		658.0	3.61		329.0	3.75	
HPV18	1639.2	1.70		409.8	3.96		204.9	3.46	
HPV31	822.1	2.50		205.5	2.74		102.8	4.61	
HPV33	738.1	2.40		184.5	2.79		92.3	4.23	
HPV45	768.2	3.21		192.0	4.32		96.0	3.60	
HPV52	816.3	2.96		204.0	3.74		102.0	4.79	
HPV58	773.2	1.66		193.3	2.91		96.6	4.74	

### 2.3 9种型别VLP稳定性分析

与铝佐剂吸附的VLP相比,未经铝佐剂吸附的VLP(即HPV原液)稳定性较差,容易出现降解等现象。将本文方法用于新生产的HPV原液中VLP的含量测定,获得HPV原液中原始的VLP浓度。将该样品于4 ℃环境下放置一周后,再次测试VLP浓度,与VLP的原始浓度相比,4 ℃放置一周后的VLP出现了明显的降解,尤其是HPV18和HPV58两种型别的VLP,降解率分别高达62.6%和41.5%。该结果表明,未经铝佐剂吸附的VLP具有热不稳定性,生产所得VLP需低温保存或尽快用铝佐剂处理。该方法灵敏度可满足原液中VLP的浓度测试和VLP稳定性测试。

Lowry法是测试蛋白质含量常用的方法。该方法通过磷钼酸试液衍生蛋白质后,测试650 nm处衍生物的吸光度,从而测试样品中蛋白质的含量。磷钼酸试液不仅可以衍生蛋白质,还可以衍生很多小分子干扰物质。大部分衍生后的小分子干扰物质会导致吸光度降低,少数衍生后的小分子干扰物质导致吸光度升高。为了降低这些干扰物质对测试结果的影响,通常需要在测试之前进行蛋白沉淀等除杂质操作。

与Lowry法相比,本方法具有自动化程度高、无小分子干扰、检测通量高等优点。

### 2.4 铝佐剂吸附后样品中游离VLP分析

铝佐剂对VLP的吸附完整性与疫苗的质量息息相关。本次共检测来自3个厂家的12批次样品,典型色谱图见图2。死时间附近可检测到明显的小分子基质,但VLP出峰位置处无明显响应。说明被检测厂家均能提供较好的铝佐剂吸附工艺,可实现VLP的良好吸附。

**图 2:**
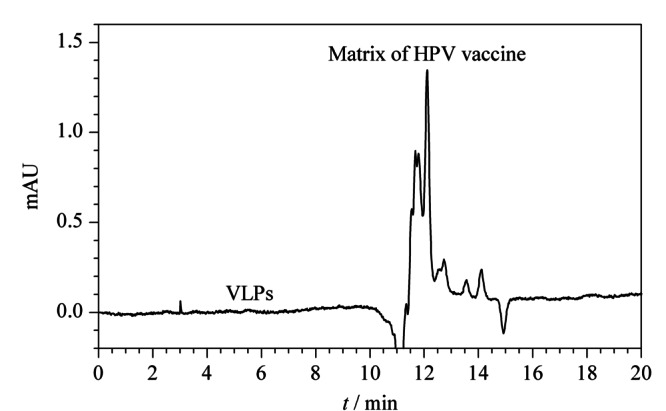
铝佐剂吸附后的VLP样品色谱图

## 3 结论

本文发展了一种基于体积排阻色谱的定量检测HPV原液中VLP的方法。该方法与Lowry法相比,具有干扰少、自动化程度高、稳定性好的优点,适合高通量的样品分析。本方法可用于HPV原液中VLP的含量测定,对比不同存储条件下HPV原液中VLP的含量变化,为探索HPV原液存储条件提供参考数据。同时,本方法还可用于疫苗上清液中游离VLP的分析,从而实现监测铝佐剂对VLP吸附的完整性。
